# Strain imaging as a prognostic indicator for complications in COVID-19 patients

**DOI:** 10.1007/s10554-024-03170-3

**Published:** 2024-07-16

**Authors:** Justin L. Devera, Choo P. Wee, Jina Sohn

**Affiliations:** 1 Division of Cardiovascular Medicine, University of California Davis, Sacramento, CA USA; 2 Division of Biostatistics, University of Southern California Keck School of Medicine, Los Angeles, CA USA; 3 Division of Cardiovascular Medicine, University of Southern California Keck School of Medicine, Los Angeles, CA USA

**Keywords:** COVID-19, Left ventricular function, Right ventricular function, Speckle-tracking echocardiography, Strain

## Abstract

The goal of this study was to determine the potential for right ventricular (RV) and left ventricular (LV) strain to predict cardiopulmonary complications of COVID-19. We identified 276 patients with COVID-19 who underwent transthoracic echocardiography within 30 days of COVID-19 diagnosis at our institution. Patients were excluded if they had a history of any primary outcomes before COVID-19 diagnosis or insufficient imaging. LV global longitudinal strain (GLS) and RV GLS were obtained using 2-dimensional speckle-tracking echocardiography. Primary outcomes were death, pulmonary embolism, congestive heart failure (CHF), cardiomyopathy, pulmonary fibrosis, pulmonary hypertension, acute respiratory distress syndrome (ARDS), and myocardial infarction (MI) occurring after COVID-19 diagnosis. In the final analysis of 163 patients, mean RV GLS and LV GLS were reduced, and 43.6% developed at least one primary outcome. There were significant differences in LV GLS distribution in terms of CHF, cardiomyopathy, and MI in bivariate analysis. However, LV GLS was not significantly associated with CHF after adjusting for LV ejection fraction and RV fractional area change, nor with MI after adjusting for troponin T. RV GLS was significantly associated with ARDS after adjusting for other variables. In the risk stratification of patients with COVID-19, strain imaging can provide incremental prognostic information, as worsened RV GLS is associated with the development of ARDS.

## Introduction

Coronavirus disease-2019 (COVID-19) caused by Severe Acute Respiratory Syndrome Coronavirus 2 (SARS-CoV2) has become a global health crisis. Although primarily a respiratory illness, COVID-19 can lead to many cardiovascular complications including myocarditis, myocardial infarction (MI), venous thromboembolism, and heart failure [1, 2]. The pathophysiology of COVID-19 is multifaceted and postulated to begin with SARS-CoV2 entry into human cells via host receptor angiotensin-converting enzyme 2 that is expressed in most tissues [1, 3]. The ensuing complications can occur at various time intervals after contagion. In 1–2 weeks, acute respiratory distress syndrome (ARDS) may develop in severe cases [4–6]. Subsequently, extrapulmonary organ involvement can manifest as cardiac injury or disseminated intravascular coagulation [7]. Alternatively, cardiac complications may develop in otherwise mild cases of illness and persist for several months beyond time of original diagnosis [8, 9]. Given the significant morbidity and mortality associated with COVID-19, it is imperative that clinicians can predict outcomes and utilize appropriate preventive strategies.

Strain analysis using 2-dimensional speckle-tracking echocardiography (2D-STE) is a technique for detecting early, subclinical changes in cardiac function [10]. So far, several studies have evaluated the use of myocardial strain obtained by 2D-STE as a means for determining prognosis of patients with COVID-19. Current data suggests that right ventricular (RV) free wall longitudinal strain and RV global longitudinal strain (GLS) predict mortality and need for intubation and mechanical ventilation in COVID-19 patients, and can outperform conventional measures of RV function such as RV fractional area change (FAC) and tricuspid annular plane systolic excursion (TAPSE) [11–18]. Similarly, left ventricular (LV) GLS has been demonstrated to be predictive of mortality, independent of RV strain [12]. However, data on the use of strain in predicting outcomes of COVID-19 can be conflicting and are overall limited, with most measuring outcomes of only mortality and/or intubation [13, 14, 17–19]. Accordingly, we sought to determine the utility of early measurement of biventricular strain in predicting individual cardiopulmonary complications of COVID-19.

## Methods

### Study population

This retrospective study was performed at Los Angeles General Medical Center (formerly known as Los Angeles County + USC Medical Center), located in Los Angeles, California and operated by Los Angeles County Department of Health Services. The hospital provides full healthcare services for the medically underserved and is one of the largest public teaching hospitals in the United States. We included a total of 276 adult patients with COVID-19 who were diagnosed according to guidance by the World Health Organization from March 16, 2020 to March 9, 2021 and who underwent echocardiography within 30 days of COVID-19 diagnosis. Patients were excluded if they had a history of any primary outcomes preceding their diagnosis of COVID-19, or suboptimal images. Of the 276 patients, 94 were excluded because they had a past medical history that included at least one outcome, and clinical data were collected for the remaining 182 patients to determine the association of non-strain parameters with outcomes. Subsequently, 19 patients were excluded because of insufficient images for strain analysis and 163 patients were included in the final analysis to determine the association of strain with clinical outcomes. The study was approved by the institutional review board of University of Southern California. This research did not receive any specific grant from funding agencies in the public, commercial, or not-for-profit sectors.

### Clinical data

Patients’ demographic characteristics, medical histories, vital signs, laboratory results, inpatient treatments, echocardiography data, and outcomes were retrieved from their electronic medical records and recorded in the database by a single analyst. Patients were reviewed for primary outcomes of pulmonary embolism (PE), congestive heart failure (CHF), cardiomyopathy, pulmonary fibrosis, pulmonary hypertension (HTN), ARDS, MI, and death occurring within 60 days after COVID-19 diagnosis. ARDS was defined as acute, diffuse lung injury with new or worsening respiratory symptoms and hypoxemia, with bilateral opacities on chest imaging that was not fully explained by cardiogenic pulmonary edema, atelectasis, or lung masses. CHF was defined by the documentation of the primary clinician’s assessment of CHF as a clinical diagnosis, with signs of fluid retention related to cardiac dysfunction as supported by elevated natriuretic peptides and/or echocardiographic findings. MI was defined as myocardial injury evidenced by high-sensitivity cardiac troponin above the 99th percentile upper reference limit with a rising and/or falling pattern, along with evidence of acute myocardial ischemia based on patient’s signs, symptoms, and electrocardiogram. Median time between COVID-19 diagnosis and echocardiography was 3 days (interquartile range: 1 to 9 days). The final follow-up date was July 2, 2021.

### Conventional echocardiographic analysis

Echocardiography data were obtained from bedside transthoracic echocardiographic examinations performed by ultrasound technicians using the Philips EPIQ CVx ultrasound system (Philips North America Corporation). Conventional echocardiographic measurements were obtained by experienced cardiology faculty in accordance with guidelines by American Society of Echocardiography (ASE) [20]. LV end-systolic volume (ESV), LV end-diastolic volume (EDV), and LV ejection fraction (EF) were measured using biplane Simpson method. LV diastolic function was assessed using the ratio of early transmitral flow velocity (E) to early diastolic medial LV septal tissue velocity (e’). Left atrial (LA) volume was calculated by obtaining biplane method of disks in apical 4- and apical 2-chamber views and indexed by body surface area [21, 22]. The average LA volume index was categorized as normal (16–34 mL/m^2^), mildly enlarged (35–41 mL/m^2^), moderately enlarged (42–48 mL/m^2^), or severely enlarged (> 48 mL/m^2^), unless otherwise specified by the reading physician due to limitations of imaging quality [22]. Right atrial (RA) volume was determined from the apical 4-chamber view and categorized as normal (≤ 27 mL/m^2^ in women, ≤ 32 mL/m^2^ in men) or enlarged (> 27 mL/m^2^ in women, > 32 mL/m^2^ in men) [23]. RV FAC, TAPSE, and lateral annular tissue velocity (S’) were also measured according to ASE guidelines [23]. Pulmonary artery systolic pressure (PASP) was determined using the peak tricuspid regurgitation (TR) jet velocity, simplified Bernoulli equation, and estimated RA pressure.

### Speckle-tracking echocardiographic analysis

Biventricular strain measurements were acquired according to recommendations by ASE and European Association of Cardiovascular Imaging and recorded as absolute values for simpler interpretation [24]. All images were analyzed using TOMTEC-ARENA TTA2 software and measurements were obtained by a single author with verification by cardiology faculty. LV GLS was measured using the apical 4-, apical 2-, and apical 3-chamber views. RV GLS was measured using the apical 4-chamber RV-focused view. After tracing the LV and RV endocardial borders, manual correction of the automatically generated region of interest was performed to achieve optimal tracking and inclusion of the myocardium, if necessary. LV GLS was automatically computed by the software as the average segmental strain components in an 18-segment model. RV GLS was automatically computed by the software as average segmental strain components for the basal, mid, and apical segments of the RV free wall and septum.

### Statistical analysis

Continuous numeral variables are expressed as mean ± standard deviation (SD) or median (interquartile range) as appropriate based on the distribution. Categorical variables are expressed as frequency (percentage). Distribution differences of LV GLS or RV GLS in terms of each outcome were measured using a two-sample t-test for normally distributed data or the Wilcoxon Rank-Sum test for non-normally distributed data. Bivariate and multivariate logistic regression analysis was performed to examine the association between biventricular strain and clinical outcomes with at least 10% incidence in the study population and occurrence of any clinical outcomes or complications. Variables with p values < 0.05 in bivariate analysis were included and adjusted for in a multivariate logistic regression model, unless the variable was not obtained or unavailable in more than 70% of the patients. The multivariate model was stratified by sex and race for those with significant non-additive effects. Receiver operating characteristic (ROC) curves were generated to evaluate the sensitivity, specificity, and optimal cutoff values (maximum Youden index) for predicting outcomes. Survival curves were derived from Kaplan-Meier analysis to assess time-to-event probabilities after COVID-19 diagnosis, based on optimal cutoff values and tertiles of strain, with comparison using the log-rank test. Logistic regression analysis was performed using Stata 17 (StataCorp, College Station, Texas) and ROC curve and survival analysis models were generated using MedCalc Statistical Software version 19.2.6 (MedCalc Software, Ostend, Belgium; https://www.medcalc.org; 2020). P values < 0.05 were considered statistically significant.

### Intra-observer reproducibility

Intra-observer variability of LV GLS and RV GLS was evaluated in 20 randomly selected patients by remeasuring values after several weeks and using intraclass correlation coefficients (ICC) and Bland-Altman analysis.

## Results

### Patient characteristics

Demographic and clinical characteristics of the 182 patients are shown in Table 1. The majority were between 41 and 70 years of age, male (68%), and Hispanic (80%). Most patients had at least one comorbidity, and hypertension and diabetes were the two most prevalent comorbidities. Median D-dimer, C-reactive protein (CRP), and mean B-type natriuretic peptide (BNP) were elevated. Mean troponin T was normal. About 19.2% of patients were receiving vasopressors and 17.6% were intubated and on mechanical ventilation at the time of echocardiography. In patients who were mechanically ventilated, the average fraction of inspired oxygen was 51% and average positive end-expiratory pressure was 7.5 mmHg. Incidence of any complications was 44%. Incidence of death was 7.4%, CHF 15.3%, ARDS 19%, and MI 19% in all patients. PE and pulmonary fibrosis were less common, occurring in 2.5% and 0.6% of all patients, respectively. The average time between the date of positive COVID-19 testing and diagnosis of ARDS was approximately 4 days, and the average interval for MI and CHF were 1 and 2 days, respectively. The average LV EF of patients diagnosed with CHF was 43% with a standard deviation of 15%.

### Echocardiographic characteristics

Median LV EF and mean E/e’, S’, TAPSE, and RV FAC were normal (Table 1). The left and right atria were of normal size in most patients. Mean LV GLS and mean RV GLS were reduced in the 163 patients who had sufficient imaging for strain analysis.

**Table 1:** Demographic and clinical characteristics (*N* = 182)

Demographics	
Age group (years)	
≤20	3 (1.65%)
21–30	17 (9.34%)
31–40	16 (8.79%)
41–50	37 (20.33%)
51–60	41 (22.53%)
61–70	40 (21.98%)
71–80	20 (10.99%)
≥ 81	8 (4.40%)
Male	124 (68.13%)
Race	
White	11 (6.04%)
Black	14 (7.69%)
Hispanic	145 (79.67%)
Asian	4 (2.20%)
Others	8 (4.40%)
BMI (kg/m^2^)	28.53 ± 7.07
**Comorbidities**	
HTN	80 (43.96%)
DM	73 (40.11%)
Obesity	54 (29.67%)
COPD	3 (1.65%)
CAD	5 (2.75%)
CKD	33 (18.13%)
**Laboratory findings**	
D-dimer (µg/mL)	2.00 (0.66–5.94)
Troponin T (ng/mL)	0 (0–0.04)
BNP (pg/mL)	1111.50 (272–5327)
CRP (mg/L)	62 (20.2–164)
**Vital signs**	
SpO2 (%)	97 (95–100)
Heart rate (beats/min)	92.48 ± 21.09
Systolic BP (mmHg)	127.76 ± 22.51
Diastolic BP (mmHg)	74.64 ± 15.25
**Treatments**	
Mechanical ventilation	32 (17.58%)
Vasopressor(s)	35 (19.23%)
Beta-blocker or non-dihydropyridine calcium channel blocker	36 (19.78%)
Anti-coagulation, therapeutically dosed	24 (13.19%)
**Left heart**	
LV EDV (mL)	99.29 ± 35.64
LV ESV (mL)	38 (27–47)
LV EF by biplane (%)	60 (55–65)
E/e’	9 (7–12)
LA volume	
Normal	143 (78.57%)
Dilated - unspecified	7 (3.85%)
Dilated - mildly	8 (4.40%)
Dilated - moderately	6 (3.30%)
Dilated - severely	3 (1.65%)
Not reported	15 (8.24%)
**Right heart**	
RA volume	
Small	108 (59.34%)
Dilated	53 (29.12%)
Not reported	21 (11.54%)
RV FAC (%)	43.24 ± 9.66
S’ (cm/s)	14.50 ± 5.09
TAPSE (cm)	2.05 ± 0.54
TR peak velocity (m/s)	2.60 ± 0.47
PASP (mmHg)	34 (26–45)
**Strain**	
LV GLS^a^ (%)	16.80 ± 5.25
RV GLS^b^ (%)	17.60 ± 7.24

Values are mean ± SD, n (%), or median (interquartile range). ^a,b^Statistical values for strain are given for subset of 163 patients and are absolute values

BMI = body mass index; BNP = B-type natriuretic peptide; BP = blood pressure; CAD = coronary artery disease; CKD = chronic kidney disease; COPD = chronic obstructive pulmonary disease; CRP = C-reactive protein; DM = diabetes mellitus; E/e’ = ratio between early mitral inflow velocity and mitral annular early diastolic velocity; EDV = end-diastolic volume; EF = ejection fraction; ESV = end-systolic volume; FAC = fractional area change; GLS = global longitudinal strain; HTN = hypertension; LA = left atrium; LV = left ventricle; PASP = pulmonary artery systolic pressure; RA = right atrium; RV = right ventricle; SD = standard deviation; SpO2 = peripheral capillary oxygen saturation; S’ = Doppler-tissue imaging-derived peak tricuspid lateral annular systolic velocity; TAPSE = tricuspid annular plane systolic excursion; TR = tricuspid regurgitation

### Bivariate analysis

Table 2 summarizes the distribution of LV GLS and RV GLS by each clinical outcome, and Table 3 shows the association of clinical data with outcomes of at least 10% incidence in the study population. There were differences in the mean of LV GLS for CHF (*p* < 0.0001), cardiomyopathy (*p* = 0.008), MI (*p* = 0.033), and any complications (*p* = 0.002), and in the mean of RV GLS in terms of ARDS (*p* = 0.001) and any complications (*p* = 0.010). Risk of any complications was increased in patients with obesity and abnormal peripheral capillary oxygen saturation (SpO2), heart rate, systolic blood pressure, D-dimer, troponin T, BNP, CRP, LV ESV, LV EF, RV FAC, and TR peak velocity.

**Table 2:** Distribution of LV GLS and RV GLS by outcomes

Variable	*N*	LV GLS	*P*	RV GLS	*P*
**Death**			0.482		0.713
No	151	16.89 ± 5.27		17.66 ± 7.39	
Yes	12	15.78 ± 5.09		16.82 ± 4.90	
**Pulmonary embolism**			0.625		0.233
No	159	16.77 ± 5.26		17.71 ± 7.12	
Yes	4	18.08 ± 5.08		13.33 ± 11.50	
**CHF**			< 0.0001		0.142
No	138	17.57 ± 4.83		17.97 ± 7.40	
Yes	25	12.57 ± 5.51		15.69 ± 6.09	
**Cardiomyopathy**			0.008		0.389
No	155	17.05 ± 5.11		17.48 ± 7.22	
Yes	8	12.04 ± 5.85		19.63 ± 7.74	
**Pulmonary fibrosis**			NA		NA
No	162	16.75 ± 5.21		17.66 ± 7.23	
Yes	1	25.9		8.33	
**Pulmonary HTN**			0.067		0.353
No	160	16.91 ± 5.18		17.68 ± 7.16	
Yes	3	11.30 ± 7.15		13.74 ± 12.37	
**ARDS**			0.304		0.001
No	132	17.01 ± 5.19		18.50 ± 7.24	
Yes	31	15.93 ± 5.50		13.82 ± 5.99	
**MI**			0.033		0.300
No	132	17.23 ± 5.02		17.90 ± 7.34	
Yes	31	15.00 ± 5.85		16.44 ± 6.81	
**Any complications**			0.002		0.010
No	92	17.92 ± 4.66		18.91 ± 7.26	
Yes	71	15.36 ± 5.63		15.97 ± 6.92	

Values of *p* < 0.05 were considered to indicate statistical significance

ARDS = acute respiratory distress syndrome; CHF = congestive heart failure; MI = myocardial infarction; NA = not applicable; other abbreviations as in Table 1.

**Table 3A:** Bivariate analysis of demographic and laboratory data with outcomes

		Any complications			CHF			ARDS			MI		
Variable	*N*	OR	95% CI	*P*	OR	95% CI	*P*	OR	95% CI	*P*	OR	95% CI	*P*
Age group^a^	182	1.19	0.99–1.43	0.067	1.02	0.79–1.31	0.883	1.34	1.04–1.71	0.021	1.26	1.00-1.58	0.050
Male	182	1.17	0.62–2.19	0.632	1.40	0.56–3.53	0.474	0.97	0.44–2.16	0.946	1.91	0.81–4.48	0.138
Race	182												
White		Ref.	Ref.		Ref.	Ref.		Ref.	Ref.		Ref.	Ref.	
Black		2.16	0.43–10.84	0.350	10.0	0.995–100.46	0.050	1.67	0.13–21.19	0.694	2.50	0.38–16.42	0.340
Hispanic		0.87	0.25–2.99	0.827	1.33	0.16–11.03	0.793	2.61	0.32–21.19	0.370	1.08	0.22–5.26	0.927
Asian		3.60	0.28–46.36	0.326	3.33	0.16–70.91	0.440	3.33	0.16–70.91	0.440	4.50	0.37–54.16	0.236
Others		0.40	0.05–2.93	0.367	1.43	0.08–26.90	0.812	NA	NA	NA	NA	NA	NA
BMI	179	1.02	0.98–1.07	0.324	1.00	0.95–1.06	0.885	1.01	0.96–1.06	0.680	1.01	0.96–1.06	0.706
Comorbidities	182												
HTN		1.18	0.65–2.13	0.581	1.02	0.45–2.33	0.956	1.81	0.85–3.83	0.123	2.54	1.21–5.33	0.014
DM		1.31	0.72–2.37	0.375	1.47	0.65–3.34	0.358	2.98	1.38–6.44	0.005	1.55	0.75–3.21	0.237
Obesity		1.95	1.02–3.71	0.042	1.48	0.63–3.49	0.366	1.89	0.87–4.10	0.107	1.37	0.64–2.95	0.416
COPD		NA	NA	NA	NA	NA	NA	2.21	0.19–25.13	0.522	8.23	0.73–93.35	0.089
CAD		0.85	0.14–5.19	0.857	1.45	0.16–13.51	0.743	NA	NA	NA	2.70	0.44–16.81	0.286
CKD		1.96	0.91–4.20	0.085	1.74	0.67–4.53	0.259	2.26	0.96–5.36	0.063	1.96	0.84–4.60	0.120
D-dimer group^b^	149	1.44	1.07–1.94	0.016	1.08	0.73–1.59	0.703	1.23	0.87–1.74	0.233	1.57	1.10–2.24	0.013
Troponin T group^c^	158	2.43	1.65–3.58	< 0.0001	1.36	0.94–1.98	0.105	1.09	0.75–1.58	0.638	3.98	2.57–6.18	< 0.0001
BNP group^d^	100	2.37	1.54–3.64	< 0.0001	2.34	1.43–3.82	0.001	1.31	0.86–1.99	0.212	3.28	1.90–5.67	< 0.0001
CRP group^e^	135	1.84	1.32–2.57	< 0.0001	1.00	0.66–1.52	0.986	2.54	1.62–3.98	< 0.0001	1.52	1.06–2.18	0.023
SpO2 group^f^	182	0.61	0.46–0.80	0.001	0.74	0.50–1.09	0.123	0.39	0.25–0.60	< 0.0001	0.69	0.49–0.98	0.036
Heart rate	182	1.02	1.004–1.03	0.009	1.00	0.98–1.02	0.750	1.01	0.99–1.03	0.403	1.01	0.99–1.03	0.310
Systolic BP	182	0.99	0.97–0.999	0.032	1.00	0.98–1.02	0.834	0.98	0.97-1.00	0.093	0.99	0.97–1.01	0.276
Diastolic BP	182	1.004	0.99–1.02	0.666	1.02	0.99–1.04	0.206	0.99	0.97–1.02	0.495	1.00	0.97–1.02	0.693

^a^Age group is treated as ordinal in ascending order: ≤20, 21–30, 31–40, 41–50, 51–60, 61–70, 71–80, ≥ 81

^b^D-dimer group is treated as ordinal in ascending order: ≤0.66, 0.67-2.00, 2.01–5.94, > 5.94

^c^Troponin group is treated as ordinal in ascending order: 0, 0.01–0.04, 0.05–0.23, > 0.23

^d^BNP group is treated as ordinal in ascending order: ≤272, 272.1-1111.5, 1111.6–5327, > 5327

^e^CRP group is treated as ordinal in ascending order: ≤20.2, 20.3–62, 63–164, > 164

^f^SpO2 group is treated as ordinal in ascending order: ≤94, 95–97, 98–99, > 99

All abbreviations and units as in Tables 1 and 2

This Table 3A shows the analysis of demographic and laboratory data. Values of p < 0.05 were considered to indicate stastical significance

**Table 3B:** Bivariate analysis of echocardiography data with outcomes

		Any complications			CHF			ARDS			MI		
Variable	*N*	OR	95% CI	*P*	OR	95% CI	*P*	OR	95% CI	*P*	OR	95% CI	*P*
LV EDV	156	1.00	0.99–1.01	0.973	1.02	1.004–1.03	0.010	0.98	0.97–0.996	0.012	1.00	0.99–1.01	0.681
LV ESV	155	1.02	1.004–1.03	0.010	1.04	1.02–1.06	< 0.0001	0.98	0.96–1.01	0.113	1.01	1.00-1.02	0.130
LV EF	178	0.92	0.88–0.95	< 0.0001	0.88	0.83–0.92	< 0.0001	1.00	0.96–1.03	0.920	0.96	0.93–0.995	0.025
E/e’	92	1.10	0.98–1.23	0.103	1.05	0.90–1.21	0.543	1.03	0.91–1.17	0.624	1.11	0.98–1.26	0.112
LA volume	167												
Normal		Ref.	Ref.		Ref.	Ref.		Ref.	Ref.		Ref.	Ref.	
Dilated-unspecified		2.00	0.43–9.35	0.379	NA	NA	NA	2.02	0.36–11.20	0.421	7.19	1.48–34.86	0.014
Dilated-mildly		4.50	0.87–23.27	0.073	4.00	0.87–18.49	0.076	NA	NA	NA	5.39	1.23–23.54	0.025
Dilated-moderately		0.75	0.13–4.26	0.746	1.33	0.15–12.21	0.799	1.01	0.11–9.14	0.993	1.08	0.12–9.78	0.947
Dilated-severely		3.00	0.26–34.05	0.375	13.33	1.14-156.23	0.039	10.1	0.87-117.15	0.064	2.69	0.23–31.31	0.428
RA volume	161												
Normal		Ref.	Ref.		Ref.	Ref.		Ref.	Ref.		Ref.	Ref.	
Dilated		1.25	0.65–2.42	0.508	1.56	0.64–3.80	0.325	0.66	0.27–1.59	0.351	1.63	0.73–3.63	0.237
RV FAC	159	0.93	0.90–0.97	< 0.0001	0.89	0.85–0.94	< 0.0001	0.97	0.93–1.01	0.106	0.96	0.92–0.998	0.039
S’	76	0.96	0.88–1.06	0.450	0.90	0.77–1.02	0.098	1.06	0.95–1.18	0.284	0.94	0.82–1.08	0.375
TAPSE	73	0.53	0.21–1.31	0.168	0.24	0.06–0.92	0.037	1.12	0.41–3.04	0.822	0.74	0.24–2.32	0.607
TR peak velocity	82	4.92	1.61–15.03	0.005	6.34	1.75–22.91	0.005	2.87	0.99–8.24	0.050	3.13	1.06–9.28	0.039
PASP	35	1.07	1.00-1.15	0.067	1.05	0.99–1.12	0.112	1.00	0.95–1.06	0.909	1.06	0.99–1.12	0.083
LV GLS	163	0.91	0.85–0.97	0.003	0.82	0.74–0.90	< 0.0001	0.96	0.89–1.04	0.303	0.92	0.85–0.99	0.035
RV GLS	162	0.94	0.90–0.99	0.012	0.95	0.90–1.02	0.142	0.90	0.84–0.96	0.001	0.97	0.92–1.03	0.299

All abbreviations and units as in Tables 1, 2, and 3A

This Table 3B shows the analysis of echocardiography data. Values of p < 0.05 were considered to indicate stastical significance

### Multivariate analysis

Multivariate logistic regression models with adjustment for other parameters are shown in Table 4. The association between LV GLS and any complications was varied by sex (interaction *p* = 0.012), but there were no differences between strain and any complications when stratified by sex. Troponin T and D-dimer, however, portended a greater risk of complications in men than in women. There were no differences in terms of race or sex in the association between strain and other outcomes (all interaction *p* > 0.05).

**Table 4A:** Multivariate analysis for any complications

Female				Male		
Variable	OR	95% CI	*P*	OR	95% CI	*P*
Strain						
LV GLS	1.21	0.97–1.51	0.092	0.97	0.82–1.16	0.762
RV GLS	0.96	0.85–1.08	0.471	0.89	0.77–1.01	0.079
Others						
Troponin T^a^	0.92	0.36–2.36	0.867	4.35	1.51–12.52	0.006
D-dimer^b^	2.71	0.97–7.57	0.058	2.12	1.13–4.01	0.020
LV EF	0.85	0.73-1.00	0.050	0.91	0.83–1.01	0.069

^a,b^Treated as ordinal in ascending order as in Table 3A

All abbreviations and units as in Tables 1, 2 and 3

This Table 4A shows analysis for any complications. Values of p < 0.05 were considered to indicate stastical significance

**Table 4B:** Multivariate analysis for individual outcomes

CHF				ARDS				MI			
Variable	OR	95% CI	*P*	Variable	OR	95% CI	*P*	Variable	OR	95% CI	*P*
Strain											
LV GLS	1.03	0.87–1.22	0.722	LV GLS	1.01	0.91–1.12	0.809	LV GLS	0.97	0.88–1.08	0.609
RV GLS	1.10	1.00-1.22	0.055	RV GLS	0.85	0.76–0.95	0.005	RV GLS	1.00	0.93–1.08	0.893
Others											
LV EDV	0.99	0.97–1.01	0.212	Age^a^	1.53	1.05–2.25	0.029	Troponin T^d^	3.69	2.31–5.91	< 0.0001
LV EF	0.84	0.77–0.92	< 0.0001	SpO2^b^	0.39	0.22–0.70	0.002				
RV FAC	0.90	0.83–0.97	0.008	CRP^c^	2.24	1.33–3.77	0.002				

^a,b,c,d^Treated as ordinal in ascending order as in Table 3A

All abbreviations and units as in Tables 1, 2 and 3

This Table 4B shows analysis for individual outcomes with = 10% incidence. Values of p < 0.05 were considered to indicate stastical significance

Neither LV GLS nor RV GLS was significantly associated with CHF when controlling for LV EDV, LV EF, and RV FAC, although CHF was associated with LV EF (odds ratio [OR]: 0.84; 95% confidence interval [CI]: 0.77–0.92; *p* < 0.0001) and RV FAC (OR: 0.90; 95% CI: 0.83–0.97; *p* = 0.008). RV GLS was predictive of ARDS even after adjustment for other variables (OR: 0.85; 95% CI: 0.76–0.95; *p* = 0.005). Age (OR: 1.53; 95% CI: 1.05–2.25; *p* = 0.029), SpO2 (OR: 0.39; 95% CI: 0.22–0.70; *p* = 0.002), and CRP (OR: 2.24; 95% CI: 1.33–3.77; *p* = 0.002) also independently predicted ARDS. Strain was not predictive of MI in a multivariate model when controlling for troponin T.

### Predictors of outcomes

**Fig. 1:**
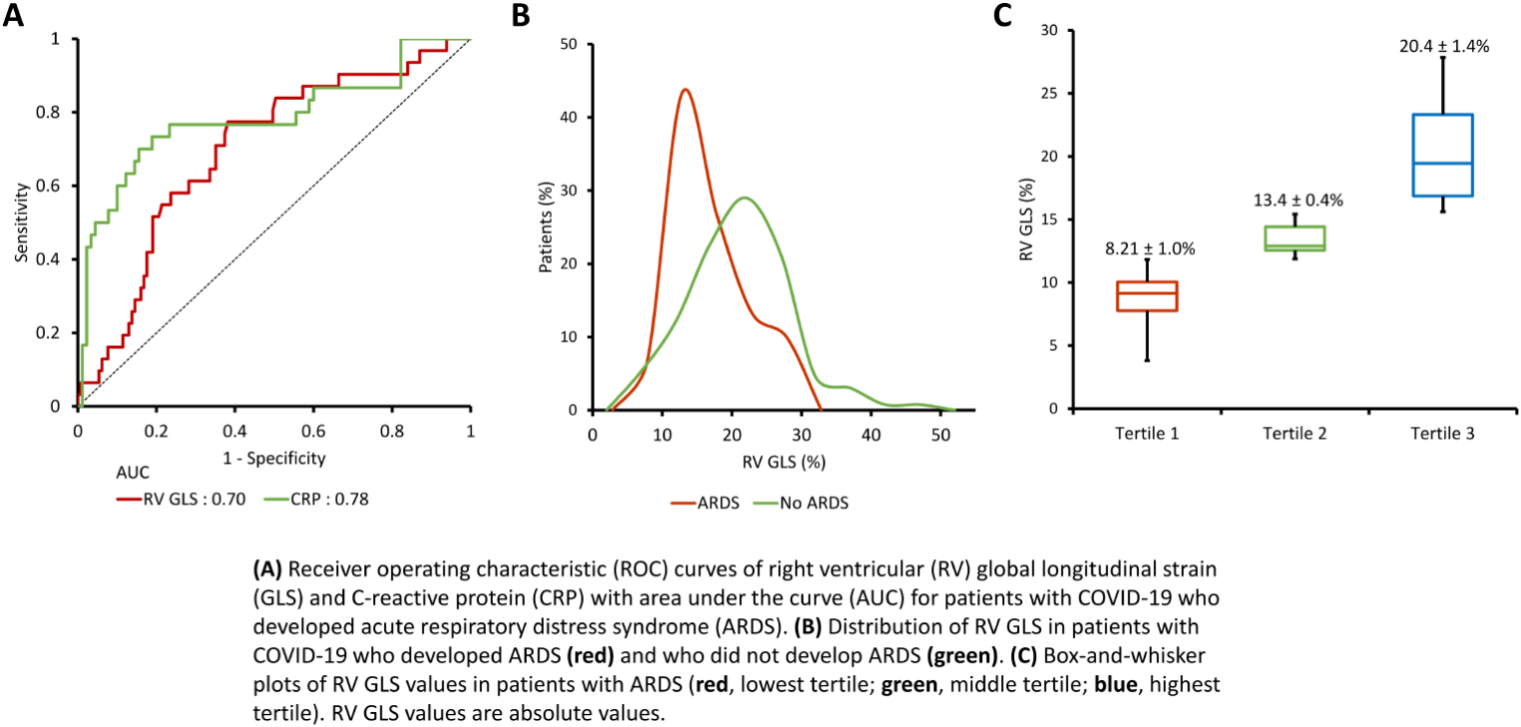
ROC curve and distribution of RV GLS for patients with ARDS

**Fig. 2:**
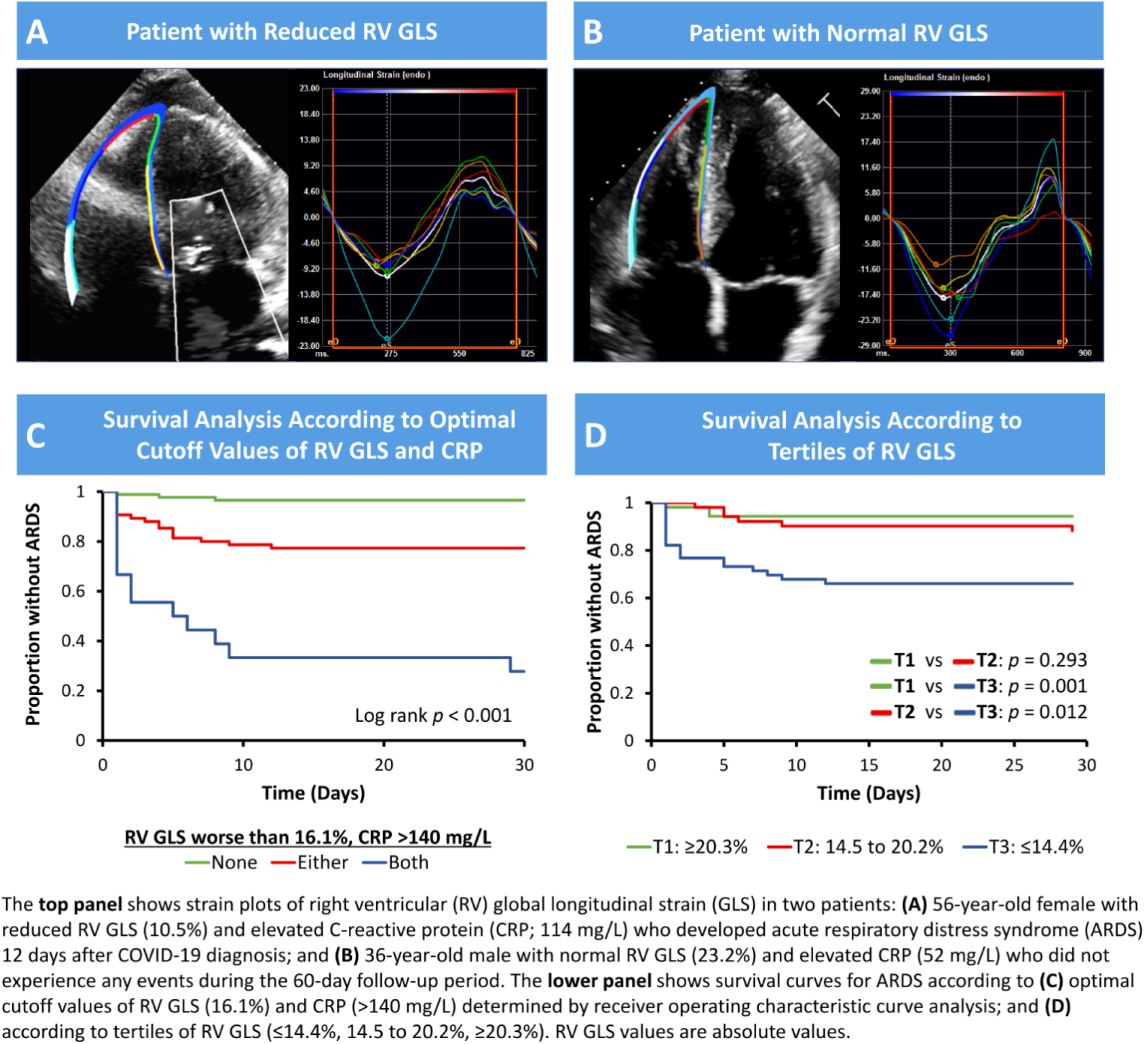
Association of RV GLS and ARDS in patients with COVID-19

**Fig. 3:**
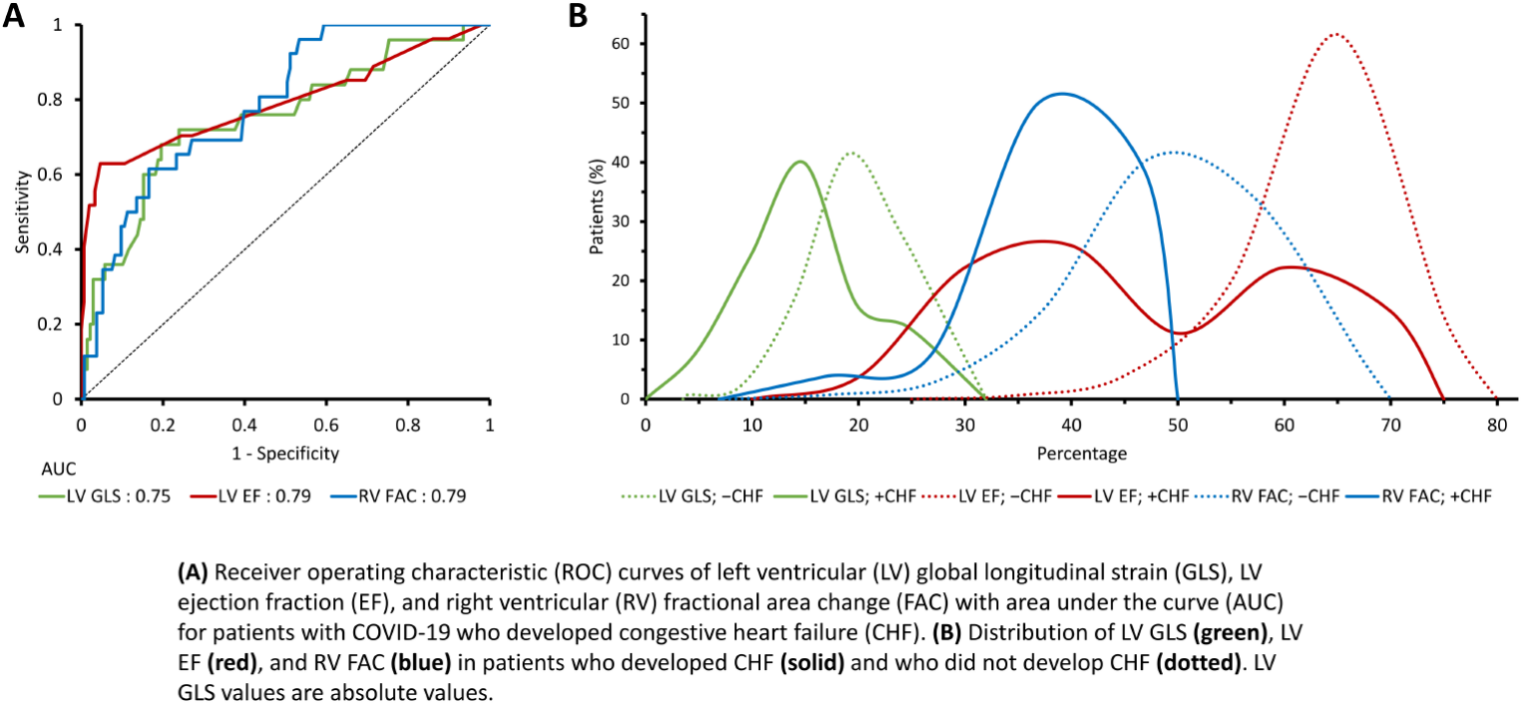
Predictors of CHF

Figure 1 shows the ROC curve for RV GLS and CRP as predictors of ARDS, and the distribution of RV GLS in those who developed ARDS. The optimal cutoff value of RV GLS and CRP for predicting ARDS was 16.12 (sensitivity 77.4, specificity 61.8) and 140 (sensitivity 70.0, specificity 84.4), respectively. Figure 2 depicts Kaplan-Maier survival curves for patients with RV GLS stratified by tertile and optimal cutoff values based on ROC analysis. The ROC curves of LV GLS, LV EF, RV FAC, as predictors of CHF are shown in Fig. 3. The area under the curve for LV EF and RV FAC were greater than that for LV GLS. Best cutoff values for predicting CHF were 45 for LV EF (sensitivity 63.0, specificity 95.4) and 36.9 for RV FAC (sensitivity 61.5, specificity 83.5).

### Reproducibility

Intra-observer reproducibility of strain was excellent. ICC was 0.996 and 0.992 for LV GLS and RV GLS, respectively. Bland-Altman analysis demonstrated small bias, with intra-observer mean difference of -0.04 and 0.24, and limits of agreement -0.90 to 0.82 and 0.90 to 1.40 for LV GLS and RV GLS, respectively.

## Discussion

To our knowledge, our study represents the first to comprehensively evaluate the potential for biventricular strain measured by 2D-STE to predict individual cardiopulmonary outcomes of COVID-19. Previous studies have investigated only either left or right ventricular strain as a predictor for mortality or need for intubation and mechanical ventilation [11–18]. We demonstrate that strain can independently predict certain outcomes of COVID-19 and early measurement may be helpful in the risk stratification of patients.

### Prognostic utility of RV GLS

We found that RV GLS was significantly associated with ARDS. Additionally, SpO2, CRP, and age were independent predictors of ARDS in a multivariate analysis. Our findings may explain those of previous studies, which demonstrate that RV strain predicts mortality and the need for intubation and mechanical ventilation, if mortality is driven by ARDS [11–18]. Early subclinical RV dysfunction may be a result of direct cardiomyocyte injury caused by SARS-CoV2, or a secondary consequence of pulmonary disease or systemic inflammation leading to RV strain and increased afterload [7, 11, 25]. TAPSE, S’, and RV FAC were not associated with ARDS, consistent with findings by Li et al. who demonstrated superiority of RV GLS to these conventional parameters in predicting COVID-19 outcomes [11]. This may be due to differences in the ability of these parameters to more completely assess RV performance, as RV GLS measures deformation throughout the entire RV free wall, in contrast to TAPSE and S’ which represent only basal longitudinal movement. Additionally, strain analysis reflects maximal and minimal values of deformation throughout the cardiac cycle, which may not be reflected in the end-systole and end-diastole frames used to measure RV FAC [11, 26, 27].

There was no association between RV GLS and CHF, although their relationship in a multivariate analysis was marginally significant (OR: 1.10, 95% CI: 1.00-1.22, *p* = 0.055) in comparison to that between LV GLS and CHF (*p* = 0.722). It is possible that our study lacked enough data to detect association between RV GLS and CHF. Regardless, the results of our data possibly suggest that right heart strain is important in the pathogenesis of CHF in COVID-19. As discussed by Bader et al. and Tajbakhsh et al., CHF in COVID-19 involves a predisposition for right-sided heart failure as a consequence of pulmonary HTN due to parenchymal lung disease and pulmonary venous thromboembolism due to a prothrombotic state [28, 29]. Otherwise, CHF due to impairment of either ventricle can result from a combination of direct viral-mediated injury, immune dysregulation, hypoxemia, and ineffective adaptation of the host’s cardiovascular system to physiologic stress induced by COVID-19 [30].

### Prognostic utility of LV GLS

Our findings suggest that LV GLS may predict cardiomyopathy. However, we were unable to rule out any potential confounders or effect modifiers in a multivariate analysis because the incidence of cardiomyopathy was less than 10%. LV GLS predicted MI in bivariate analysis, but not after adjusting for troponin T. This is consistent with prior studies demonstrating strong correlations between troponin levels and major cardiovascular events due to COVID-19 [31–34]. Additionally, we found that patients with increased CRP have higher risk of MI (OR: 1.52, 95% CI: 1.06–2.18, *p* = 0.023), suggesting that greater inflammatory burden is correlated with myocardial injury and ischemia. From a supply-demand perspective, it is unclear if COVID-19 affects the coronary arteries in a specific pattern to explain why LV GLS but not RV GLS was associated with MI in our study.

CHF was predicted by LV GLS, LV EF, and RV FAC in a bivariate analysis, but not by LV GLS when controlling for the latter two. This suggests a bimodal distribution of illness severity, wherein patients with mild infection have subclinical ventricular dysfunction and generally do not develop CHF. Conversely, those with severe infection are more prone to experiencing early, substantial cardiac involvement with greater risk of CHF.

### Clinical implications

The risk stratification of patients with COVID-19 should involve a comprehensive assessment and can be augmented by early measurement of strain to predict ARDS. Echocardiographic measurements should be considered in conjunction with other prognostic factors, including patient comorbidities, inflammatory burden, and cardiac biomarkers. When interpreting strain in patients who are intubated and receiving mechanical ventilation, clinicians should consider the effects of high ventilatory pressures on ventricular performance, as positive pressure ventilation can increase strain [35]. Accordingly, lung-protective strategies to minimize barotrauma and subsequent ventricular dysfunction may be implemented. With enhanced tools for risk stratification, more research will be needed to determine their use in directing specific cardio-protective interventions.

### Study limitations

Our study is limited by lack of a control group, baseline comparison data of echocardiography parameters, and estimation of inter-rater reliability. Additionally, strain measurements may have been influenced by mechanical ventilation at the time of echocardiography. We also cannot exclude any delays in the diagnosis or recognition of these cardiopulmonary complications of COVID-19 such that they may have already been ongoing at the time of echocardiography. Our study included asymptomatic patients with COVID-19 and was performed in a single center with a predominantly underserved and Hispanic population, which might limit generalizability [36]. Moreover, many of our patients had clinical features associated with severe illness, including being male and overweight, and having hypertension and diabetes [37–39]. Given the diversity of illness due to COVID-19, studies with multi-center involvement and larger sample sizes are needed to further ascertain the prognostic value of strain, though inter-vendor variability in 2D-STE software may impede generalizability of findings. Finally, it may be impossible to know the true latency period between contagion and development of complications from COVID-19, as the onset of symptoms and exact timing of infection could have preceded when patients actually presented for COVID-19 testing. Future research might also elucidate if COVID-19 is associated with specific, regional patterns of strain abnormalities, and the extent to which strain in COVID-19 survivors can improve [40, 41].

## Conclusion

In this retrospective study of early strain measurement in patients with COVID-19, RV GLS was found to be independently associated with ARDS. LV GLS was significantly associated with cardiomyopathy in a bivariate analysis, although this relationship could not be further evaluated in a multivariate model due to low incidence. LV GLS was predictive of CHF and MI in a bivariate analysis, but not in multivariate models controlling for LV EF and RV FAC, and troponin T, respectively.

## Data Availability

No datasets were generated or analysed during the current study.

## Abbreviations

ARDS: Acute respiratory distress syndrome
BMI: Body mass index
BNP: B-type natriuretic peptide
BP: Blood pressure
CAD: Coronary artery disease
CHF: Congestive heart failure
CI: Confidence interval
CKD: Chronic kidney disease
COPD: Chronic obstructive pulmonary disease
CRP: C-reactive protein
DM: Diabetes mellitus
E/e’: Ratio between early mitral inflow velocity and mitral annular early diastolic velocity
EDV: End-diastolic volume
EF: Ejection fraction
ESV: End-systolic volume
FAC: Fractional area change
GLS: Global longitudinal strain
HTN: Hypertension
iqr: Interquartile range
LA: Left atrium
LV: Left ventricle
MI: Myocardial infarction
NA: Not applicable
OR: Odds ratio
PASP: Pulmonary artery systolic pressure
RA: Right atrium
RV: Right ventricle
S’: Doppler-tissue imaging-derived peak tricuspid lateral annular systolic velocity
SD: Standard deviation
SpO2: Peripheral capillary oxygen saturation
TAPSE: Tricuspid annular plane systolic excursion
TR: Tricuspid regurgitation
